# Associations between psychedelic-related and meditation-related variables: A longitudinal study

**DOI:** 10.1016/j.jpsychires.2025.03.025

**Published:** 2025-03-17

**Authors:** Otto Simonsson, Sankalp Chaturvedi, Peter S. Hendricks, Cecilia U.D. Stenfors, Walter Osika, Jayanth Narayanan, Roman Palitsky, Simon B. Goldberg

**Affiliations:** a Department of Neurobiology, Care Sciences and Society, Karolinska Institutet, Stockholm, Sweden; b Center for Healthy Minds, University of Wisconsin – Madison, Madison, WI, USA; c Department of Management and Entrepreneurship, Imperial College London, London, UK; d Department of Psychiatry and Behavioral Neurobiology, University of Alabama at Birmingham, Birmingham, AL, USA; e Department of Psychology, Stockholm University, Stockholm, Sweden; f Department of Management & Organizational Development, Northeastern University, Boston, MA, USA; g Emory Center for Psychedelics and Spirituality, Emory University, Atlanta, GA, USA; h Department of Psychiatry and Behavioral Sciences, Emory University School of Medicine, Atlanta, GA, USA; i Emory Spiritual Health, Woodruff Health Sciences Center, Emory University, Atlanta, GA, USA; j Department of Counseling Psychology, University of Wisconsin - Madison, Madison, WI, USA

**Keywords:** Psychedelics, Meditation, Mindfulness, Self-compassion, Adverse, Harm

## Abstract

Previous research has investigated associations between psychedelic experiences and meditation practice, but knowledge gaps remain. Using a longitudinal research design with a sample of US residents between 18 and 50 years old (N = 13,012), we investigated associations between psychedelic-related and meditation-related variables. The follow-up survey was completed by 7484 respondents, of whom 336 reported psychedelic use during the two-month study. In covariate-adjusted regression models, psychedelic use was associated with greater increases in the number of days of mindfulness and loving-kindness or compassion meditation practice in the past week, especially among those with no prior experience of psychedelics or meditation. Among those who reported psychedelic use, trait mindfulness and trait self-compassion at baseline were associated with less severe challenging psychedelic experiences, as well as lower odds of psychedelic-occasioned thoughts or attempts of self- or other-harm. However, among those who practiced meditation at baseline, psychedelic use was associated with greater increases in past-week frequency of loving-kindness or compassion meditation-related difficulties and impairments. Future research is warranted.

## 1. Introduction

Recent decades have seen a surge in therapeutic research on psychedelics and meditation (Nutt and Carhart-Harris, 2021; Goldberg and Davidson, 2024). For example, psychedelics such as psilocybin and lysergic acid diethylamide (LSD) have shown promise in treating various psychiatric disorders when combined with psychotherapy (Yao et al., 2024). Similarly, meditation practices such as mindfulness meditation and loving-kindness or compassion meditation have demonstrated potential as mental health treatments (Goldberg et al., 2022a; Zheng et al., 2023). While these interventions have shown relative safety in controlled settings (Farias et al., 2020; Hinkle et al., 2024), recent research suggests that the risks associated with psychedelics and meditation are not fully understood and warrant further investigation (Palitsky et al., 2024; Simonsson et al., 2024a; Van Dam et al., 2018), particularly in naturalistic settings (Goldberg et al., 2022b; Lindahl et al., 2017; Evans et al., 2023; Simonsson et al., 2023b; Simonsson and Fisher, 2024; Simonsson and Goldberg, 2023).

Previous experimental research indicates that psychedelic use may, under certain conditions, have beneficial effects on meditation practice (e.g., increasing meditation depth) in experienced meditators (Berit et al., 2024; Smigielski et al., 2019). Such findings suggest potential benefits for individuals with extensive mental training, but recent observational studies involving samples more representative of the adult population (i.e., not limited to experienced meditators) have also begun to investigate associations between psychedelic use and meditation practice. For instance, in a recent longitudinal study, psychedelic use during the two-month study period was associated with a greater increase in the number of past-week days of mindfulness meditation practice (Simonsson et al., 2024b). This study did not find a significant association between psychedelic use and changes in the number of past-week days of loving-kindness or compassion meditation practice, but the sample size of participants who used psychedelics during the study period was relatively small (n = 100; Simonsson et al., 2024b; see also Simonsson et al., 2023a), potentially leading to insufficient statistical power.

Another study with 953 participants, of whom 536 reported previous meditation experience, evaluated associations of psychedelic-related and meditation-related variables cross-sectionally. This study found that lifetime psychedelic use was associated with lower perceived barriers to meditation practice, higher perceived efficacy of meditation practice, and awakening or enlightenment as motivation for meditation practice (Simonsson and Goldberg, 2023). No associations were found between psychedelic use and meditation-related adverse effects (e.g., persistent anxiety, sleeping difficulties, or disconnection; Simonsson and Goldberg, 2023), but it might have been valuable to investigate potential moderators of this link such as adverse childhood experiences and psychiatric symptoms, which have been associated with meditation-related adverse effects (Goldberg et al., 2022b).

Other research indicates that psychedelic use, under certain conditions, may also positively influence psychological constructs that are central to mindfulness meditation and loving-kindness or compassion meditation (e.g., trait mindfulness, trait self-compassion). For example, various studies have reported increases in trait mindfulness and trait self-compassion shortly after psychedelic use. However, results have been mixed at longer-term follow-ups and many these studies have lacked a control group (e.g., Domínguez-Clavé et al., 2022; Madsen et al., 2020; Sampedro et al., 2017; Smigielski et al., 2019; Soler et al., 2016, 2018). It is therefore important to further investigate whether psychedelic use may indeed be associated with sustained increases in trait mindfulness and trait self-compassion, especially days, weeks, or even months following psychedelic use, and to conduct these studies with adequately large samples.

It is entirely possible that psychedelic use and meditation practice have bidirectional effects and work has also begun to investigate whether meditation practices can impact psychedelic experiences and related variables. Recent research suggests that certain meditation practices may be associated with less severe challenging psychedelic experiences (Simonsson et al., 2024b; but see also, Johnstad, 2021), but such associations need replication and little is known about how these meditation practices might reduce the likelihood of challenging psychedelic experiences. Both mindfulness-based and loving-kindness or compassion-based interventions have been shown to increase trait mindfulness and trait self-compassion (Goldberg et al., 2019; Golden et al., 2021; Petrovic et al., 2024; Reilly and Stuyvenberg, 2023), which are constructs that could potentially explain the negative association between meditation practice and psychologically difficult states during psychedelic experiences. For instance, trait mindfulness could possibly help individuals avoid judging or resisting challenging mental states during psychedelic experiences, while self-compassion may potentially provide *internal* psychological support during difficult psychedelic experiences. Although these mental traits could, hypothetically, aid in the resolving of challenging psychedelic experiences, there is currently limited evidence to support this idea.

### 1.1. Current study

Given the limited evidence regarding how psychedelic experiences and meditation practices – and in particular the qualities they cultivate – influence one another, we conducted a longitudinal observational study with a large sample of US adults (N = 13,012) to investigate potential associations between psychedelic-related and meditation-related variables. We hypothesized that, compared with respondents who did not report psychedelic use during the two-month study period, participants who reported psychedelic use during the study period would have (1) a greater increase in past 7 days practice of mindfulness meditation, (2) a greater increase in past 7 days practice of loving-kindness or compassion meditation, (3) a greater decrease in perceived barriers to meditation, (4) a greater increase in trait mindfulness, and (5) a greater increase in trait self-compassion. We also hypothesized that among participants who reported psychedelic use during the two-month study period, (6) higher past 7-day mindfulness meditation practice at baseline, (7) higher past 7-day loving-kindness or compassion meditation practice at baseline, (8) higher trait mindfulness at baseline, and (9) higher trait self-compassion at baseline would be associated with less severe challenging psychedelic experiences. We conducted exploratory analyses to investigate potential moderators, as well as associations between psychedelic use and other related outcomes (e.g., perceived efficacy of meditation practice, motivation for meditation practice, meditation-related difficulties or impairments. We also conducted exploratory analyses to investigate associations between meditation-related variables (e.g., past-week days of practice) and constructs (e.g., trait mindfulness) at baseline and the duration of challenging, difficult, or distressing psychedelic experiences and thoughts or attempts of self-harm or harm to others following psychedelic experiences.

## 2. Method

### 2.1. Participants

The study (hypotheses, design plan, sampling plan, variables, and analysis plan) was preregistered on the Open Science Framework (OSF) at https://osf.io/rchwa and https://osf.io/3rbp5. Based on estimates from previous research, we used purposive sampling and recruited US residents between 18 and 50 years old to increase the likelihood that we recruited participants who would report psychedelic use during the study period. The participants were recruited through Prolific Academic (https://prolific.com), which is an online platform that facilitates participant recruitment.

### 2.2. Materials and procedure

From June 2023 until September 2023, participants were invited to complete a baseline survey. The same participants were invited to complete a follow-up survey approximately two months after they had completed the baseline survey. This study was part of a larger survey and completion of the baseline survey resulted in $2.50 payment and completion of the follow-up survey resulted in $2.50 payment. The recruitment materials neither mentioned psychedelics nor meditation to limit self-selection bias. Informed consent was obtained from participants. Study procedures were determined to be exempt from review by the Institutional Review Board at the University of Wisconsin-Madison.

### 2.3. Measures

#### 2.3.1. Psychedelic use

At baseline, all participants were asked to report which, if any, of the following psychedelics they had ever used: ayahuasca, N, N-dimethyltryptamine (DMT), LSD, mescaline, peyote, San Pedro, and psilocybin. If the participants reported lifetime psychedelic use at baseline, they were also asked if they had used any of these psychedelics in the past two months. At follow-up, the participants were again asked if they had used psychedelics in the past two months (i.e., during the two-month study period since the baseline survey). If the participants reported past two-month psychedelic use at follow-up, they were also asked to report the dose (low, moderate, large, very large, extreme) and extra-pharmacological factors (i.e., “set and setting”; items derived from Simonsson et al., 2023b) associated with their most intense psychedelic experience in the same time period. Those participants who reported past two-month psychedelic use at follow-up were also asked to look back on their most intense psychedelic experience in the past two months and complete the Challenging Experiences Questionnaire (CEQ; Barrett et al., 2016), which was designed to capture psychologically difficult states during the acute psychedelic experience (i.e., grief, fear, death, insanity, isolation, physical distress, paranoia). The responses were rated on a 0- (“None; not at all”) to 5-point (“Extreme”) Likert scale and the total score was calculated as the average score of all items. Internal consistency was excellent (alpha = .97 using unimputed sample at follow-up). Looking back on their most intense experience using a psychedelic in the past two months, the same participants were also asked to rate the duration of challenging, difficult, or distressing experiences during that time (0 = Not at all, 100 = All the time) and to report whether they experienced any thoughts or attempts of harming themselves or others in the days or weeks following that experience (0 = No, 1 = Yes).

#### 2.3.2. Demographics and other substance use

At baseline, all participants were asked to report age, gender identity, educational attainment, degree of religiosity, political affiliation, adverse childhood experiences (Ford et al., 2014), and personal history of any DSM-5 diagnosis (e.g., Depressive Disorders). All participants were also asked to report which, if any, of the following substances they had ever used: alcohol, nicotine products (e.g., cigarettes, e-cigarettes, cigarillos, little cigars, smokeless tobacco), cannabis products (e.g., weed, THC, CBD, hemp oil), MDMA, major stimulants (e.g., cocaine, methamphetamine), illicit narcotic analgesics/opioids (e.g., morphine, heroin, oxycodone), illicit benzodiazepines and barbiturates (e.g., Valium, Alprazolam [Xanax]), inhalants (poppers, whip-its, nitrous oxide, glue), and other substances. At follow-up, the participants were asked if they had used any of the same substances in the past two months (i.e., during the two-month study period since the baseline survey).

#### 2.3.3. Meditation practice

At baseline, all participants were asked to report whether they had ever tried mindfulness meditation. If the participants reported having tried mindfulness meditation, they were asked to estimate their total lifetime number of hours of mindfulness meditation practice. Those who reported having tried mindfulness meditation were also asked to report on how many days they engaged with mindfulness meditation over the past week. If the participants reported not having tried mindfulness meditation, they were coded as 0 days. The participants who reported having practiced mindfulness meditation 1 day or more in the past week were also asked to report, with reference to the past 7 days, how effective they had found their mindfulness meditation practice (not at all effective, slightly effective, moderately effective, very effective, extremely effective), how much their mindfulness meditation practice had been motivated by the goal of achieving awakening or enlightenment (not at all, slightly, moderately, very, completely), whether they had challenging, difficult, or distressing experiences as a result of their mindfulness meditation practice (never, rarely, occasionally, regularly, frequently), and whether such experiences impaired their ability to function in daily life (never, rarely, occasionally, regularly, frequently). At follow-up, participants were asked to report on how many days they engaged with mindfulness meditation over the past week, how effective they had found their mindfulness meditation practice, how much their mindfulness meditation practice had been motivated by the goal of achieving awakening or enlightenment, whether they had challenging, difficult, or distressing experiences as a result of their mindfulness meditation practice, and whether such experiences impaired their ability to function in daily life. Equivalent items were presented to participants in regard to loving-kindness or compassion meditation.

#### 2.3.4. Perceived barriers to meditation practice

At baseline and follow-up, all participants were asked to complete the 12-item Determinants of Meditation Practice Inventory-Revised (DMPI-R; Hunt et al., 2020), which consists of four aspects of perceived meditation barriers (i.e., perceived benefit, perceived knowledge, perceived pragmatic barriers, perceived sociocultural conflict). The responses were rated on a 1- (“Strongly disagree”) to 5-point (“Strongly agree”) Likert scale and the total score was calculated as the average score of all items, with higher total score values indicating greater perceived barriers to meditation practice. Internal consistency was good (alpha = .82 using sample at baseline).

#### 2.3.5. Trait mindfulness and self-compassion

At baseline and follow-up, all participants were asked to complete the 15-item Five Facets of Mindfulness (Gu et al., 2016), which consists of five aspects of trait mindfulness (i.e., observing, describing, acting with awareness, non-judging of inner experience, and non-reactivity to inner experience). The responses were rated on a 1- (“Never or very rarely true”) to 5-point (“Very often or always true”) Likert scale and the total score was calculated as the average score of the subscales, with higher total score values indicate greater trait mindfulness. Internal consistency was good (alpha = .78 using sample at baseline).

At baseline and follow-up, all participants were also asked to complete the 12-item Self-Compassion Scale – Short Form (Raes et al., 2011), which consists of five aspects of trait self-compassion (i.e., self-kindness, self-judgment, common humanity, isolation, mindfulness, over-identification). The responses were rated on a 1- (“Almost never”) to 5-point (“Almost always”) Likert scale and the total score was calculated as the average score of the subscales, with higher total score values indicate greater self-compassion. Internal consistency was good (alpha = .88 using sample at baseline).

#### 2.3.6. Statistical analyses

As specified in the preregistrations, we used multiple linear regression models to assess whether there were significant differences on dependent variables between those who reported psychedelic use in the two-month study period versus those who did not. These models focused on change (i.e., from baseline to follow-up) on past 7 days practice of mindfulness meditation, change on past 7 days practice of lovingkindness or compassion meditation, perceived barriers to meditation practice change scores, trait mindfulness change scores, and trait self-compassion scores, controlling for age (recoded as: 18–24, 25–34, 35–44, 45–50), gender (recoded as: male, female, other), educational attainment (Bachelor’s degree or higher, no Bachelor’s degree), religiosity (not at all religious, a little religious, moderately religious, quite religious, very religious), political affiliation (Democratic Party, Republican Party), past two month use of alcohol, nicotine products, cannabis products, MDMA, major stimulants, illicit narcotic analgesics/opioids, illicit benzodiazepines and barbiturates, inhalants, and other substances at follow-up (all substances entered as separate covariates), and psychedelic use in the past two months at baseline. Sensitivity analyses specifically for change on past 7 days of meditation practice were conducted using zero-inflated negative binomial models. In exploratory analyses, we used the same models and focused on mindfulness meditation and loving-kindness or compassion meditation with regards to change on past 7 days perceived efficacy of meditation practice, change on past 7 days awakening or enlightenment as meditation practice motivation, change on past 7 days meditation-related difficulties, and change on past 7 days meditation-related impairments. Because adverse childhood experiences and psychiatric symptoms have been associated with a higher occurrence of meditation-related adverse effects (Goldberg et al., 2022b), we conducted additional exploratory analyses and used the same models to investigate whether reporting personal history at baseline of one or more adverse childhood experiences or one or more DSM-5 diagnoses moderated the associations between psychedelic use during the study period and past 7 days meditation-related difficulties and impairments. These exploratory analyses also controlled for change on past 7 days practice of [mindfulness, loving-kindness or compassion] meditation.

As was also specified in the preregistrations, we used multiple linear regression models to assess whether past 7 days practice of mindfulness meditation at baseline, past 7 days practice of loving-kindness or compassion meditation at baseline, trait mindfulness at baseline, trait self-compassion at baseline, one or more adverse childhood experiences at baseline, or one or more DSM-5 diagnoses at baseline were associated with less severe challenging psychedelic experiences among participants who used psychedelics during the two-month study period, controlling for age, gender, educational attainment, religiosity, political affiliation, lifetime use of psychedelics, alcohol, nicotine products, cannabis products, MDMA, major stimulants, illicit narcotic analgesics/opioids, illicit benzodiazepines and barbiturates, inhalants, and other substances, and dose used during participants’ most intense psychedelic experience during the study period. The past-7-days-of-practice (but not trait mindfulness or trait self-compassion) models also controlled for lifetime hours of [mindfulness, loving-kindness or compassion] meditation practice. In exploratory analyses, we used the same models and focused on the duration of challenging, difficult, or distressing experiences during the study period’s most intense psychedelic experience while logistic regression was used for any thoughts or attempts to harm oneself or others in the days or weeks following the most intense psychedelic experience. Given the relatively early stage of research on associations between psychedelic use and meditation practices, we did not apply a p-value adjustment (e.g., Bonferroni) to correct for multiple comparisons. While such corrections are important to limit the risk of Type I errors, they can also be overly conservative and potentially obscure meaningful patterns in novel areas of research with significant public health relevance. There were no missing data at baseline and missing data at follow-up were handled by using Multivariate Imputation by Chained Equations (MICE; Van Buuren and Groothuis-Oudshoorn, 2011; mice package version 3.15.0 in R Studio) to impute the missing data across twenty imputed data sets using random forest imputations. We split the dataset into subsets based on missing-by-design structures (e.g., survey items only asked to participants who reported past-week meditation practice) and independently imputed each subset. Results were pooled using Rubin’s (1976) rules (i.e., across imputations and pooled using the ‘pool’ function in the ‘mice’ package; Van Buuren and Groothuis-Oudshoorn, 2011). The ‘scale’ function was used on all continuous variables to produce results in standardized units; odds ratios were calculated and reported for the logistic regression models. Descriptive statistics show non-imputed values only while only imputed results are presented for the multiple linear regression models.

## 3. Results

The survey at baseline was completed by 13,012 participants, of whom 7484 completed the follow-up survey two months later (i.e., 58 % retention rate). There were 336 participants who reported psychedelic use during the two-month study period (approximately 4 % of the follow-up survey completers). At baseline, there were 2019 participants who reported having practiced mindfulness meditation 1 day or more in the past week while 907 participants reported having practiced lovingkindness or compassion meditation 1 day or more in the past week.

Table 1 shows sample characteristics at baseline of respondents who reported psychedelic use during the study period (i.e., users) and respondents who did not report psychedelic use (i.e., non-users; see Table S1 for sample characteristics of full sample). As shown in the table, participants who reported psychedelic use during the study period and participants who did not report psychedelic use were significantly different with regard to age category, gender identity, religiosity, political affiliation, and previous drug use. Among those who reported psychedelic use, the majority reported psilocybin use, as well as a low or moderate dose, during the most intense psychedelic experience in that time period and almost half of those who used psychedelics during the study reported that the most intense psychedelic experience in the same period was associated with no preparation, a major life event prior to the experience, a negative mindset, no psychological support, a disagreeable physical environment, or a too large dose.

Table 2 displays results from confirmatory analyses testing the associations between psychedelic-related and meditation-related variables (see Table S2 for results from exploratory analyses). Psychedelic use during the study period was associated with a greater increase in the number of days of both mindfulness and loving-kindness or compassion meditation practice in the past week, especially among those with no prior experience of psychedelics or meditation. However, no significant associations were observed between psychedelic use during the study period and change in perceived barriers to meditation, trait mindfulness, or trait self-compassion. Among those who reported psychedelic use during the study period, trait mindfulness and trait self-compassion at baseline were associated with less severe challenging experiences during the most intense psychedelic experience throughout the study time period, as well as lower odds of thoughts or attempts to harm oneself or others in the days or weeks following the most intense psychedelic experience. However, no significant associations were observed between past-week mindfulness meditation practice or loving-kindness or compassion meditation practice at baseline or personal history of one or more adverse childhood experiences reported at baseline and severity or duration of challenging experiences during the most intense psychedelic experience, nor thoughts or attempts to harm oneself or others in the days or weeks following the most intense psychedelic experience. Notably, personal history of any DSM-5 diagnosis was associated with more severe challenging experiences during the most intense psychedelic experience throughout the study time period, as well as higher odds of thoughts or attempts to harm oneself or others in the days or weeks following the most intense psychedelic experience. Among those who practiced meditation at baseline, psychedelic use during the study period was associated with increases in past-week frequency of lovingkindness or compassion meditation-related difficulties and impairments, but no significant associations were observed between psychedelic use during the study period and past-week frequency of mindfulness meditation-related difficulties or impairments, past-week perceived efficacy of mindfulness meditation practice or lovingkindness or compassion meditation practice, or past-week awakening or enlightenment motivation for mindfulness meditation practice or loving-kindness or compassion meditation practice. No interactions between personal history of adverse childhood experiences or DSM-5 diagnosis and psychedelic use during the study period on meditation-related difficulties and impairments were observed. The results were broadly the same in sensitivity analyses and imputation did not meaningfully change results compared with non-imputed data.

## 4. Discussion

This longitudinal observational study investigated associations between psychedelic-related and meditation-related variables. The results showed that psychedelic use during the study period was associated with a greater increase in the number of days of both mindfulness and lovingkindness or compassion meditation practice in the past week, especially among those with no prior experience of psychedelics or meditation. Among those who reported psychedelic use during the study period, trait mindfulness and trait self-compassion were both associated with less severe challenging psychedelic experiences, as well as lower odds of psychedelic-related thoughts or attempts of self- or other-harm. However, among those who practiced meditation at baseline, psychedelic use during the study period was associated with a greater increase in past-week frequency of loving-kindness or compassion meditation-related difficulties and impairments.

As hypothesized, psychedelic use during the study period was associated with a greater increase in the number of days of both mindfulness and loving-kindness or compassion meditation practice in the past week. These increases could potentially be explained by a psychologically insightful psychedelic experience prompting meditation practice as part of broader health behavior changes (e.g., Teixeira et al., 2022), a challenging psychedelic experience prompting meditation as part of coping strategies (e.g., Robinson et al., 2024a, 2024b), or other factors (e.g., spiritual exploration). Other confirmatory analyses showed that only higher trait mindfulness and trait self-compassion at baseline were associated with less severe challenging psychedelic experiences, even though the past-week meditation practice items were correlated with trait mindfulness and trait self-compassion (see Table S3). Such findings suggest that these traits may be predictive of acute and post-acute psychedelic-related outcomes, which should be investigated further in future research.

Contrary to our hypotheses, no significant associations were observed between psychedelic use during the study period and change in perceived barriers to meditation, trait mindfulness, or trait self-compassion. Such null findings may have been the result of extra-pharmacological factors, especially given the high percentage of psychedelic users who reported extra-pharmacological factors (e.g., no psychological support) that have previously been associated with more severe challenging psychedelic experiences (Simonsson et al., 2023b). Previous research with psychedelic administration paired with psychotherapy, for example, does appear to have produced increases in trait self-compassion among those with alcohol use disorder (Agin-Liebes et al., 2023), which may not necessarily have increased without the psychotherapy component (Gründer et al., 2024). Future research should explore potential interactions between psychedelic use and extra-pharmacological factors on psychological constructs related to meditation (e.g., trait self-compassion).

In exploratory analyses, psychedelic use during the study period was not significantly associated with change in past-week perceived efficacy of meditation practice, past-week awakening or enlightenment motivation for meditation practice, or past-week frequency of mindfulness meditation-related difficulties and impairments. However, psychedelic use in that period was associated with a greater increase in past-week frequency of loving-kindness or compassion meditation-related difficulties and impairments. Because the effects of psychedelics and certain meditation practices share neurophysiological and phenomenological overlaps (Millière et al., 2018), it is not unreasonable to expect certain overlaps in lasting psychedelic- and meditation-related difficulties and impairments. It is possible therefore that psychedelic use might increase, for example, self-perception alterations such as depersonalization under certain circumstances (Evans et al., 2023), which could be amplified by continued meditation practice (Britton et al., 2021). If the findings in this study related to loving-kindness or compassion meditation-related difficulties and impairments are replicated in future studies, it would be important to understand the mechanisms underlying the relationships between psychedelic use and meditation-related adverse effects, as well as the type of meditation-related adverse effects that may be associated with psychedelic use.

## 5. Limitations

This study has a number of limitations. First, we used purposive sampling to recruit participants who would be likely to use psychedelics during the study period. The findings may not be generalizable to the broader population, although it is worth noting that the recruitment materials did not mention psychedelics or meditation, which may have limited self-selection of participants with favorable attitudes toward these topics. Second, the retention rate at follow-up was 58 % and multiple imputation was used to address the missing data. The results may have been biased, however, if the assumptions underlying the imputation process were incorrect. Third, neither the past-week days of meditation practice response items (i.e., 0–7) nor the psychedelic dose response items (i.e., low – extremely large) were especially specific. This may have led to different interpretations of what they meant across participants. Fourth, the items on psychedelic-related and meditation-related adverse outcomes generally lacked specificity also. For instance, one item asked participants whether they had challenging, difficult, or distressing experiences as a result of their meditation practice in the past week. If participants would have been presented with a more specific item (e.g., whether they had depersonalization experiences as a result of their meditation practice in the past week), the study could potentially have provided more insight into the specific ways in which psychedelic use may lead to meditation-related difficulties. Fifth, due to the observational study design used in this study, no conclusive causal inferences can be made. Future studies should utilize randomized controlled research designs to evaluate the potential relationships between psychedelic-related and meditation-related variables.

## Supplementary Material

Multimedia component 1

## Figures and Tables

**Table 1:** Sample characteristics.

	Psychedelic use during the study period		
	Yes (n = 336)	No (n = 7148)	*p*

**Age**			.018
18–24	24 %	19 %	
25–34	44 %	41 %	
35–44	24 %	28 %	
45–50	9 %	12 %	
**Gender identity**			<.001
Male	49 %	38 %	
Female	45 %	57 %	
Other	7 %	5 %	
**Education**			.253
Bachelor’s degree or higher	52 %	56 %	
Less than bachelor’s degree	48 %	44 %	
**Religious belief**			.026
Not at all religious	58 %	49 %	
A little religious	18 %	21 %	
Quite religious	12 %	15 %	
Moderately religious	7 %	9 %	
Very religious	5 %	6 %	
**Political affiliation**			.009
Democratic Party	82 %	76 %	
Republican Party	18 %	24 %	
**Lifetime substance use**			
Psychedelics	81 %	21 %	<.001
Alcohol	87 %	76 %	<.001
Nicotine products	74 %	48 %	<.001
Cannabis products	87 %	62 %	<.001
MDMA	48 %	14 %	<.001
Major stimulants	48 %	16 %	<.001
Illicit narcotic analgesics or opioids	25 %	11 %	<.001
Illicit benzodiazepines and barbiturates	36 %	14 %	<.001
Inhalants	32 %	9 %	<.001
Other substances	14 %	4 %	<.001
**Personal history of adverse childhood experiences**			
One or more reported	91 %	80 %	<.001
**Personal history of DSM-5 diagnoses**			
One or more reported	67 %	55 %	<.001
**Substance during most intense psychedelic experience**			
Ayahuasca	3 %	…	…
DMT	8 %	…	…
Psilocybin	66 %	…	…
LSD	24 %	…	…
Mescaline	9 %	…	…
Peyote	2 %	…	…
San Pedro	1 %	…	…
**Dose during most intense psychedelic experience**			
Low	33 %	…	…
Moderate	48 %	…	…
Large	13 %	…	…
Very large	4 %	…	…
Extremely large	2 %	…	…
**Set and setting of most intense psychedelic experience**			
Negative mindset prior to the experience	18 %	…	…
No psychological support during the experience	17 %	…	…
Disagreeable physical environment	13 %	…	…
Insufficient preparation for the experience	10 %	…	…
Disagreeable social environment	9 %	…	…
Major life event prior to experience	8 %	…	…
Combining a classic psychedelic with another drug	7 %	…	…
Disagreeable musical environment	6 %	…	…
Dose was too large	4 %	…	…
Other	3 %	…	…

Note: This table shows sample characteristics at baseline of respondents who reported psychedelic use during the study period and respondents who did not report psychedelic use. All percentages, which represent distributions within psychedelics users and non-users, were rounded to the nearest 1%; cumulative percentages may not add to 100.0. The items with psychedelic substance type, as well as set and setting, were multiple-choice. Pearson’s chi-squared tests were used to examine the characteristics of users versus non-users.

**Table 2:** Regression estimates - confirmatory analyses.

Independent variables	Dependent variables	Coef(SE)	*p*

Psychedelics use	MM practice change score	0.23 (0.07)	<.001
Psychedelics use	LKCM practice change score	0.24 (0.07)	<.001
Psychedelics use	Perceived barriers to meditation change score	0.03 (0.07)	.622
Psychedelics use	Trait mindfulness change score	0.00 (0.07)	.991
Psychedelics use	Trait self-compassion change score	−0.01 (0.07)	.906
MM practice at baseline	Severity of challenging psychedelic experiences	−0.02 (0.05)	.715
LKCM practice at baseline	Severity of challenging psychedelic experiences	− 0.01 (0.05)	.880
Trait mindfulness at baseline	Severity of challenging psychedelic experiences	−0.12 (0.05)	.001
Trait self-compassion at baseline	Severity of challenging psychedelic experiences	−0.12 (0.05)	.002

Note: MM = mindfulness meditation; LKCM = loving-kindness or compassion meditation. Coef = standardized units (i.e., continuous variables were z-scored using the ‘scale’ function); standard errors within brackets. Change score = baseline to follow-up.

## Data Availability

The data and script are available at https://figshare.com/s/20d6e3f2923b8fa17f38.
