# Supplementary material for: Associations between psychedelic-related and meditation-related variables: A longitudinal study
Source: J Psychiatr Res. Author manuscript; Available in PMC 2025 Apr 15. (PMC11999004; doi:10.1016/j.jpsychires.2025.03.025)
Supplement: Multimedia component 1 [file NIHMS2068637-supplement-Multimedia_component_1.docx]

Supplemental Materials

| Table S1. Sample characteristics of full sample (N=13,012) | |
| --- | --- |
| Age |  |
| 18-24 | 25% |
| 25-34 | 42% |
| 35-44 | 24% |
| 45-50 | 10% |
| Gender identity |  |
| Male | 37% |
| Female | 58% |
| Other | 5% |
| Education |  |
| Bachelor’s degree or higher | 55% |
| Less than bachelor’s degree | 45% |
| Religious belief |  |
| Not at all religious | 49% |
| A little religious | 22% |
| Quite religious | 15% |
| Moderately religious | 8% |
| Very religious | 5% |
| Political affiliation |  |
| Democratic Party | 77% |
| Republican Party | 23% |
| Lifetime substance use |  |
| Psychedelics | 24% |
| Alcohol | 78% |
| Nicotine products | 50% |
| Cannabis products | 65% |
| MDMA | 15% |
| Major stimulants | 18% |
| Illicit narcotic analgesics or opioids | 11% |
| Illicit benzodiazepines and barbiturates | 14% |
| Inhalants | 10% |
| Other substances | 4% |
| Adverse childhood experiences |  |
| One or more reported | 82% |
| Personal history of any DSM-5 diagnosis |  |
| One or more reported | 57% |

| Table S2. Regression Estimates – Exploratory Analyses | | | | |
| --- | --- | --- | --- | --- |
| Independent variables | Dependent variables | Coef (SE) | aOR (95% CI) | *p* |
| Psychedelics use | MM practice: perceived efficacy change score | 0.05 (0.12) |  | .697 |
| Psychedelics use | MM practice: enlightenment motivation change score | 0.07 (0.11) |  | .514 |
| Psychedelics use | MM practice: difficulties change score | 0.18 (0.12) |  | .144 |
| Psychedelics use | MM practice: impairments change score | 0.16 (0.13) |  | .213 |
| Psychedelics use | LKCM practice: perceived efficacy change score | 0.08 (0.17) |  | .642 |
| Psychedelics use | LKCM practice: enlightenment motivation change score | 0.08 (0.16) |  | .622 |
| Psychedelics use | LKCM practice: difficulties change score | 0.34 (0.17) |  | .048 |
| Psychedelics use | LKCM practice: impairments change score | 0.34 (0.17) |  | .045 |
| Psychedelic use x Past meditation use | MM practice change score | -0.46 (0.11) |  | <.001 |
| Psychedelic use x Past meditation use | LKCM practice change score | -0.46 (0.12) |  | <.001 |
| Psychedelic use x Past psychedelic use | MM practice change score | -0.53 (0.15) |  | <.001 |
| Psychedelic use x Past psychedelic use | LKCM practice change score | -0.74 (0.15) |  | <.001 |
| Psychedelic use x ACE ≥ 1 | MM practice: difficulties change score | 0.21 (0.35) |  | .552 |
| Psychedelic use x ACE ≥ 1 | MM practice: impairments change score | 0.53 (0.34) |  | .136 |
| Psychedelic use x ACE ≥ 1 | LKCM practice: difficulties change score | 0.76 (0.41) |  | .066 |
| Psychedelic use x ACE ≥ 1 | LKCM practice: impairments change score | 0.65 (0.42) |  | .125 |
| Psychedelic use x DSM-5 ≥ 1 | MM practice: difficulties change score | 0.37 (0.24) |  | .126 |
| Psychedelic use x DSM-5 ≥ 1 | MM practice: impairments change score | 0.30 (0.24) |  | .209 |
| Psychedelic use x DSM-5 ≥ 1 | LKCM practice: difficulties change score | 0.34 (0.33) |  | .303 |
| Psychedelic use x DSM-5 ≥ 1 | LKCM practice: impairments change score | 0.14 (0.32) |  | .670 |
| ACE ≥ 1 | Severity of challenging psychedelic experiences | 0.02 (0.16) |  | .886 |
| DSM-5 ≥ 1 | Severity of challenging psychedelic experiences | 0.20 (0.10) |  | .042 |
| MM practice at baseline | Duration of challenging psychedelic experiences | 0.03 (0.05) |  | .575 |
| LKCM practice at baseline | Duration of challenging psychedelic experiences | -0.06 (0.06) |  | .309 |
| Trait mindfulness at baseline | Duration of challenging psychedelic experiences | -0.08 (0.05) |  | .090 |
| Trait self-compassion at baseline | Duration of challenging psychedelic experiences | -0.08 (0.05) |  | .090 |
| ACE ≥ 1 | Duration of challenging psychedelic experiences | -0.08 (0.17) |  | .647 |
| DSM-5 ≥ 1 | Duration of challenging psychedelic experiences | 0.11 (0.10) |  | .286 |
| MM practice at baseline | Attempts or thoughts of harm to self or others |  | 0.59 (0.34, 0.96) | .056 |
| LKCM practice at baseline | Attempts or thoughts of harm to self or others |  | 1.10 (0.61, 2.03) | .759 |
| Trait mindfulness at baseline | Attempts or thoughts of harm to self or others |  | 0.52 (0.32, 0.81) | .005 |
| Trait self-compassion at baseline | Attempts or thoughts of harm to self or others |  | 0.39 (0.22, 0.65) | <.001 |
| ACE ≥ 1 | Attempts or thoughts of harm to self or others |  | 0.80 (0.22, 3.16) | .737 |
| DSM-5 ≥ 1 | Attempts or thoughts of harm to self or others |  | 5.86 (2.09, 19.12) | .002 |
| Note: MM = mindfulness meditation; LKCM = loving-kindness or compassion meditation. Coef = standardized units; standard errors within brackets. Change score = baseline to follow-up. Past meditation use = Ever used [mindfulness, loving-kindness or compassion] meditation reported at baseline; past psychedelic use = ever used psychedelics reported at baseline; ACE ≥ 1 = one or more adverse childhood experiences; DSM-5 ≥ 1 = one or more DSM-5 diagnoses. | | | | |

| Table S3. Correlations Between Meditation Practice and Trait Variables | | | |
| --- | --- | --- | --- |
| Variables | | Spearman’s rho | p |
| Past-week MM | Trait Mindfulness | .11 | <.001 |
| Past-week MM | Trait Self-Compassion | .13 | <.001 |
| Past-week LKCM | Trait Mindfulness | .06 | <.001 |
| Past-week LKCM | Trait Self-Compassion | .12 | <.001 |
| Note: MM = mindfulness meditation; LKCM = loving-kindness or compassion meditation. | | | |
